# Supervisor’s Negative Mood and Healthcare Workers’ Voice Behavior: A Moderated Mediation Model

**DOI:** 10.3389/fpsyg.2021.761527

**Published:** 2022-01-20

**Authors:** Ping Yuan, Yuan Cheng, Yanbin Liu, Shifeng Liu

**Affiliations:** ^1^Logistics and E-Commerce College, Zhejiang Wanli University, Ningbo, China; ^2^School of Public Affairs, Zhejiang University, Hangzhou, China; ^3^Business School, NingboTech University, Ningbo, China

**Keywords:** constructive voice, defensive voice, negative mood, mood contagion, healthcare workers

## Abstract

Healthcare workers’ voice is of importance in decreasing medical accidents and improving the efficacy of hospital units. To investigate the impact and the underlying mechanisms of supervisors’ negative mood on healthcare workers’ voice behavior, based on the mood contagion perspective, we designed a cross-sectional study, with 299 healthcare workers from mainland China completed the questionnaires. The results indicated supervisors’ negative mood was positively related to healthcare workers’ negative mood, which further led to less constructive voice and more defensive voice. Moreover, the healthcare worker’s emotional intelligence aspect of self-emotion appraisal moderated this relationship, but not others-emotion appraisal. We believe healthcare workers’ supervisors should pay attention to their negative mood expression and regulation. In the hope of reducing being influenced by supervisors’ negative mood, training about noticing and recognizing their own emotions are needed for healthcare workers.

## Introduction

In response to the increasing uncertainty and rapid changes caused by the COVID-19 pandemic, healthcare workers in hospital units are expected to be more proactive about their work (Chen et al., 2021). *Voice behavior* is a proactive behavior defined as “informal and discretionary communication by an employee of ideas, suggestions, concerns, information about problems, or opinions about work-related issues…” (Morrison, 2014, p. 174) that has been found to benefit organizations by enhancing work efficacy, reducing risks, and improving the work process (Nemeth, 1997; Weick and Sutcliffe, 2001; Lam and Mayer, 2014). Because hierarchies often tend to hinder employees of lower status from voicing out, researchers have emphasized the role of team supervisors in encouraging subordinates to engage in voice behavior (e.g., Detert and Treviño, 2010). For example, the supervisor’s leadership style (Detert and Burris, 2007), behavior (Edmondson, 2003; Nembhard and Edmondson, 2006; Tangirala and Ramanujam, 2012; Weiss et al., 2018), relation with subordinates (Ilies et al., 2007), and positive mood (Liu et al., 2015). However, efforts are still needed to fully understand the whole picture of how the characteristics of supervisors would influence subordinators’ voice behavior, especially in medical settings.

First, research about the influence of supervisors’ negative moods on voice behavior is limited. Moods are “generalized feeling states of relatively low intensity with no clear antecedent causes” (Sy et al., 2005, p. 295). Moods are different from emotions, which are short-lived affective states associated with specific events (Frijda, 1986). Moods can serve as a social cue that influences people’s behaviors in interpersonal relationships (Van Kleef et al., 2010). Especially in the Chinese cultural context with the existence of power distance, the behaviors of employees are likely to incline toward their supervisor’s mood (Liu et al., 2015). Moreover, in healthcare work settings, negative moods are common (Szczygiel and Mikolajczak, 2018), and are quite intensified by the pandemic (Stamu-O’Brien et al., 2020; Clark, 2021; Lyons et al., 2021; Nemţeanu et al., 2021; Platt, 2021). Moreover, supervisors’ negative moods have also been found to have an impact on employees’ attitudes and behavior (e.g., Glomb and Hulin, 1997; Sy et al., 2005). Thus, it is worthwhile to investigate the influence of supervisors’ negative moods on healthcare workers’ voice behavior in medical organizations. Second, previous research has explored the underlying mechanisms of supervisors’ mood on employees’ voice from the perspective of the employees’ psychological or cognitive state, such as a state of psychological safety (Liu et al., 2015), but not from their own mood states. According to the mood contagion perspective (Neumann and Strack, 2000), we assume that supervisors’ mood can also impact employees’ behavior by influencing their mood. For example, a supervisor’s negative mood might lead employees to have a negative mood. Additionally, negative mood triggers more risk-avoidance attitudes and behaviors (e.g., Mittal and Ross, 1998), and engaging in voice behavior is often considered to be risky (Morrison, 2014). Thus, when employees have supervisors in negative moods, they might have less constructive suggestions and more defensive opinions. Third, current research and researchers have called for specifying the boundary conditions and contextual factors that influence voice behavior, but most research focused on the external or interpersonal factors (e.g., Maynes and Podsakoff, 2014; Li et al., 2017). Thus, in this study, we focus on the personal characteristics that might interact with supervisor’s mood and healthcare workers: based on the mood contagion perspective, we believe healthcare workers’ emotional intelligence, that is, the ability to appraise their own and others’ emotions, could play moderating roles in the association between supervisors’ mood and healthcare workers’ voice behavior.

In sum, the main purpose of this study is to investigate the role of the supervisor’s negative mood in impacting healthcare workers’ voice behavior and examine the underlying and boundary mechanism in this association. Our findings would have several important implications for the field. Theoretically, we contribute to the voice behavior literature by investigating the role and the underlying mechanism of supervisors’ negative moods on employees’ voice behavior through the perspective of mood contagion. Empirically, this study was conducted in hospital units and focused on the voice behavior of healthcare workers, which help to enlarge our understanding of the voice behavior of specific subjects in certain contexts. Practically, with the boundary effect of healthcare worker’s emotional intelligence in our proposed theoretical model, insights were given in the managemental practices in protecting and encouraging healthcare worker’s voice behavior.

### Supervisors’ Negative Mood and Healthcare Workers’ Voice

According to the mood contagion perspective, people tend to have a congruent mood states through the observation of another individual’s public display of mood (Neumann and Strack, 2000). Hatfield et al. (1993) proposed that individuals are likely to transmit their mood when they are able to express their mood, and individuals are likely to receive others’ mood when they are able to attend to others’ moods and understand the expressed moods. In healthcare work settings, from a supervisor’s perspective, they are able to control and influence group members’ resources, time and work interactions, so they may have more opportunities to express and transmit their mood; from a subordinate’s perspective, as they depend more on their supervisor because of hierarchy, they are more likely to attend to their supervisor’s mood. Consistent with this reasoning, supervisors’ moods have an impact on employees’ moods (Sy et al., 2005). Specifically, supervisors’ negative mood will lead to their employees having similar negative moods, for example, fear, depressed mood and sadness (Sy et al., 2005; Van Kleef et al., 2009).

Employees’ negative mood also impacts their voice behavior (e.g., Hsiung and Tsai, 2017). Maynes and Podsakoff (2014) defined voice behavior as “an individual’s voluntary and open communication directed toward individuals within the organization that is focused on influencing the context of the work environment” (p. 88), and they also divided voice behavior into different dimensions including constructive voice and defensive voice: constructive voice intends to effect organizational changes in the work context, and defensive voice is about opposition to changes in policies and practices in an organization, even when the proposed changes are necessary. As voice behavior changes the *status quo* of the organization, it is commonly considered a challenging behavior that carries personal risks (Morrison, 2014), and a negative mood often leads to risk avoidance attitudes and behaviors (Mittal and Ross, 1998). Thus, healthcare workers in a negative mood tend to keep opinions about changes or develops about organizations to themselves, that is, engage in less constructive voice, and try to decrease changes in the work environment and procedures, that is, engage in more defensive voice.

Based on the above reasoning, supervisors’ negative mood positively influences healthcare workers’ negative mood, and healthcare workers’ negative mood subsequently leads to less constructive voice and more defensive voice. We predict the following:

*Hypothesis 1: Healthcare workers’ negative mood mediates the association of supervisors’ negative mood and their constructive voice.*
*Hypothesis 2: Healthcare workers’ negative mood mediates the association of supervisors’ negative mood and their defensive voice.*

### Moderating Role of Emotional Intelligence and the Moderated Mediation

On the basis of the mood contagion perspective, healthcare workers’ emotional intelligence might play a role in the association between supervisors’ moods and their moods. Emotional intelligence refers to one’s ability to monitor and recognize one’s own and others’ feelings and emotions and use this information to guide one’s thoughts and behaviors (Salovey and Mayer, 1990), it is also closely related to people’s ability to perceive and manage their own and others’ moods (e.g., Ciarrochi et al., 2000). Law et al. (2004) divided emotional intelligence into four dimensions: self-emotion appraisal (SEA), others-emotion appraisal (OEA), use of emotion (UOE), and regulation of emotion (ROE). This research focuses on the perceptions of others’ emotions and experiences of their own emotions; thus, we assessed the SEA and OEA dimensions of emotional intelligence in this study: SEA describes people’s ability to understand and express their deep emotions, and OEA refers to the ability to perceive and understand the emotions of the people around them.

We assume that SEA and OEA play different moderating roles in the contagious process between supervisors’ negative mood and healthcare workers’ negative mood. When healthcare workers have stronger SEA, they tend to sense and acknowledge their emotions better than others (Law et al., 2004), which might lead to a clear distinction between others’ mood and their own mood; because of this, supervisors’ mood may have a small impact on their mood. When healthcare workers have stronger OEA, they will have greater recognition of others’ moods (Law et al., 2004), so they can perceive their supervisors’ moods more precisely. Thus, their supervisors’ negative mood may have greater impact on their own negative mood.

In summary, we predict:

*Hypothesis 3: Healthcare workers’ SEA negatively moderates the association of supervisors’ negative mood and their negative mood such that the association is weaker when healthcare workers’ SEA is higher.*
*Hypothesis 4: Healthcare workers’ OEA positively moderates the association of supervisors’ negative mood and their negative mood such that the association is stronger when healthcare workers’ OEA is higher.*

Taking hypothesis 3 and hypothesis 4 together, we predict that healthcare workers’ SEA and OEA also have different influences on the impact of supervisors’ negative mood on different aspects of voice through healthcare workers’ negative mood. Specifically, healthcare workers with stronger SEA have more understanding of their own moods and thus are more able to differentiate their mood from supervisors, which can lead to less mood contagion from their supervisors’ negative mood and less impact on their voice behavior.

For healthcare workers with stronger OEA, as they can sense and perceive their supervisor’s mood more strongly and precisely, they will have a more negative mood than those with weaker OEA, which can further lead to less constructive voice and more defensive voice.

Thus, we predict the following:

*Hypothesis 5: Healthcare workers’ SEA negatively moderates the indirect effect by which supervisors’ negative mood influences constructive voice through the mediating role of healthcare workers’ negative mood, such that the indirect effect is weaker when healthcare workers’ SEA is higher.*
*Hypothesis 6: Healthcare workers’ OEA positively moderates the indirect effect by which supervisors’ negative mood influences constructive voice through the mediating role of healthcare workers’ negative mood, such that the indirect effect is stronger when healthcare workers’ OEA is higher.*

**FIGURE 1:**
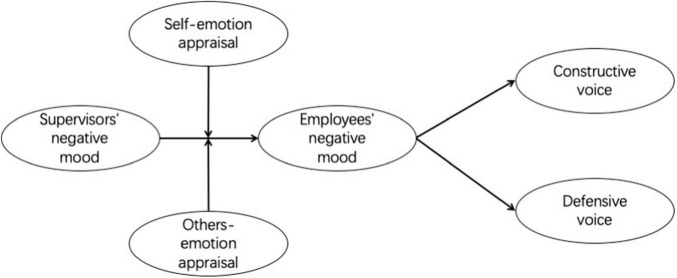
The proposed model.

Figure 1 illustrates our conceptual model.

## Materials and Methods

### Design and Participants

This study has a cross-sectional design. Links of the online questionnaire were distributed in the online work groups of hospitals in the northern part of mainland China. A sample of 299 healthcare workers (doctors and nurses) was recruited. Among those, 184 were female (61.5%), the average age was 38.15 years old (*SD* = 6.05), the average tenure was 14.33 years (*SD* = 7.0), 70.9% had a technical degree or bachelor’s degree, and the remaining 29.1% had a master’s or doctoral degree.

### Measurements

All measurements that did not have Chinese versions were translated and verified by a standard translation and back-translation procedure (Brislin, 1980).

Constructive voice and defensive voice were measured by the voice measure from Maynes and Podsakoff (2014), and each concept contains five items. Five-point Likert-type scales (1 = strongly disagree; 5 = strongly agree) were used. A sample item for constructive voice was “Often suggests changes to work projects in order to make them better,” and its Cronbach’s α coefficient was 0.93. A sample item for defensive voice was “Vocally opposes changing how things are done, even when changing is inevitable,” and its Cronbach’s α coefficient was 0.94.

The supervisors and healthcare worker negative moods that were selected were “distressed,” “angry,” “upset” and “irritated.” Five-point Likert-type scales (1 = strongly disagree; 5 = strongly agree) were used. A sample item for supervisors’ negative mood was “In the past 1 month, I feel my supervisor is distressed,” and its Cronbach’s α coefficient was 0.89. A sample item for healthcare workers’ negative mood was “In the past 1 month, I feel myself distressed,” and its Cronbach’s α coefficient was 0.91.

Self- and other- emotion appraisal. SEA and OEA were measured by the subscales of the Chinese version (Liu et al., 2011) of Law et al. (2004) Wong and Law Emotional Intelligence scale. Each dimension contains 4 items, and 7-point Likert-type scales (1 = strongly disagree; 7 = strongly agree) were used. A sample item for SEA was “I really understand what I feel,” and its Cronbach’s α coefficient was 0.94. A sample item for OEA was “I am a good observer of others’ emotions,” and its Cronbach’s α coefficient was 0.95.

Control variables: Participants’ age, gender, education level and tenure were measured to control for possible unrelated effects, as outlined in LePine and Van Dyne (1998). Information on gender was gathered by the following response categories: 0 = male, 1 = female; for education level: 1 = middle school degree or below, 2 = high school, 3 = technical degree or bachelor’s degree, 4 = master’s or doctoral degree^1^.

### Procedures

The participants were asked to complete the study measures. This study was approved by the ethics committee of the first author’s institution. Participants were informed that their responses were kept confidential and only be used for research purposes and that they can discontinue their participation at any moment. At the end of the study, participants were thanked and debriefed.

## Results

Confirmatory factor analysis presented a satisfactory fit between the observed data and the hypothesized model (χ^2^ = 149.96, *df* = 120, CFI = 0.99, TLI = 0.99, RMSEA = 0.03, SRMR = 0.03) indicating good discriminative validity of the variables. As we hypothesized, supervisors’ negative mood was positively related to healthcare workers’ negative mood (γ = 0.32, *p* < 0.001) and negatively related to healthcare workers’ constructive voice (γ = –0.33, *p* < 0.001), but it was positively related to defensive voice (γ = 0.18, *p* < 0.01). Meanwhile, healthcare workers’ negative mood was negatively related to their constructive voice (γ = –0.41, *p* < 0.001) and positively related to their defensive voice (γ = 0.21, *p* < 0.001). See Table 1.

**TABLE 1:** Descriptive and correlations.

Variables	Mean	*SD*	1	2	3	4	5	6	7	8	9
1. Age	38.015	6.05									
2. Gender	0.38	0.49	0.30**								
3. Education level	3.29	0.46	0.13*	0.21**							
4. Work tenure	14.33	7.00	0.89**	0.21**	–0.11						
5. Supervisors’ negative mood	2.85	0.98	0.01	0.13*	0.02	–0.03					
6. Healthcare worker’s negative mood	2.85	1.07	–0.08	0.10	–0.04	–0.10	0.32**				
7. Constructive voice	3.53	0.95	0.00	–0.10	–0.02	0.04	−0.33**	−0.41**			
8. Defensive voice	2.84	1.07	0.02	0.17**	–0.05	0.03	0.18**	0.21**	−0.18**		
9. SEA	4.91	1.09	–0.03	0.11	–0.10	0.05	0.02	−0.17**	0.10	–0.05	
10. OEA	2.53	0.54	–0.07	–0.02	–0.05	–0.04	0.00	0.03	0.13*	−0.12*	0.51**

*N = 299. SEA, self-emotion appraisal; OEA, others-emotion appraisal.*

**p < 0.01, *p < 0.05, two-tailed.

Mplus 7.0 (Muthén and Muthén, 2002) was used to test the mediation and moderation effects. The results indicated that there were significant indirect effects of supervisors’ negative mood on constructive voice (β = –0.11, *p* < 0.01) and defensive voice (β = 0.06, *p* < 0.05) through healthcare workers’ negative mood. We further examined the mediation effect using the Monte Carlo method (Preacher and Selig, 2012). For the indirect effect on constructive voice, a 95% CI for 20,000 simulated samples did not include zero [CI (–0.17, –0.04)]; for the indirect effect on defensive voice, a 95% CI for 20,000 simulated samples also did not include zero [CI (–0.002, 0.12)]. Thus, hypothesis 1 and hypothesis 2 were confirmed (see Table 2).

**TABLE 2:** The direct effects and indirect effects.

Variable	Model 1	Model 2	Model 3
	Healthcare worker’s negative mood	Constructive voice	Defensive voice
**Direct effects**			
Supervisor’s negative mood	0.35**	–0.32**	0.19**
Healthcare worker’s negative mood		–0.30**	0.17*
**Indirect effects**			
Supervisor’s negative mood → Healthcare worker’s negative mood → Constructive voice			–0.11, CI [–0.17, –0.04]
Supervisor’s negative mood → Healthcare worker’s negative mood → Defensive voice			0.06, CI [–0.002, 0.12]

N = 299. **p < 0.01, *p < 0.05, two-tailed.

Hypothesis 3 and hypothesis 4 predicted moderating roles of healthcare workers’ SEA and OEA in the relation between supervisors’ negative mood and healthcare workers’ negative mood. Our results showed that for healthcare workers with high SEA (1 *SD* higher than the average), the negative effect of supervisors’ negative mood on healthcare workers’ negative mood was significant (β = –0.36, *p* < 0.001), while for healthcare workers with low SEA (1 *SD* lower than the average), this association was not statistically significant (β = 0.05, *p* > 0.05). In addition, for healthcare workers with high OEA (1 *SD* higher than the average), the effect of supervisors’ negative mood on healthcare workers’ negative mood was not significant (β = 0.06, *p* > 0.05); for healthcare workers with low OEA (1 *SD* lower than the average), the abovementioned effect was not significant (β = 0.08, *p* > 0.05). Thus, hypothesis 3 was supported, but hypothesis 4 was not.

Hypothesis 5 and hypothesis 6 examined the moderating effect of healthcare workers’ SEA and OEA on the indirect effect of supervisors’ negative mood on healthcare workers’ voice through healthcare workers’ negative mood. The results showed that based on the SEA dimension: regarding the healthcare workers’ constructive voice, the indirect effect of *supervisors’ negative mood—healthcare workers’ negative mood—healthcare workers’ constructive voice* was significant for healthcare workers with high SEA (1 *SD* higher than the average), γ = 0.13, *p* < 0.001, while this indirect effect was not significant for healthcare workers with low SEA (1 *SD* lower than the average), γ = –0.02, *p* > 0.05*;* regarding their defensive voice, the indirect effect of *supervisors’ negative mood—healthcare workers’ negative mood—healthcare workers’ defensive voice* was significant for healthcare workers with high SEA (1 *SD* higher than the average), γ = –0.08, *p* < 0.05, while this indirect effect was not significant for healthcare workers with low SEA (1 *SD* lower than the average), γ = 0.01, *p* > 0.05. Based on the OEA dimension, the following was found: Regarding healthcare workers’ constructive voice, the indirect effects of *supervisors’ negative mood—healthcare workers’ negative mood—healthcare workers’ constructive voice* were both not significant for healthcare workers with high OEA (1 *SD* higher than the average), γ = –0.02, *p* > 0.05, and for healthcare workers with low OEA (1 *SD* lower than the average), γ = –0.03, *p* > 0.05*;* regarding their defensive voice: the indirect effects of *supervisors’ negative mood—healthcare workers’ negative mood—healthcare workers’ defensive voice* were both not significant for healthcare workers with high OEA (1 *SD* higher than the average), γ = 0.01, *p* > 0.05, and for healthcare workers with low OEA (1 *SD* lower than the average), γ = 0.02, *p* > 0.05. Thus, hypothesis 5 was supported, but hypothesis 6 was not.

## Discussion

From the mood contagion perspective, this study aimed to explore the influence of supervisors’ negative mood on healthcare workers’ voice behavior, with the mediating role of healthcare workers’ negative mood and the moderating role of healthcare workers’ emotional intelligence: SEA and OEA. This study makes several contributions to the field.

Theoretically, we demonstrated the utility of viewing supervisors’ negative moods as one antecedent of healthcare workers’ voice behavior. This result responds to the call for theory building and empirical work on the role of affect in voice behavior (Morrison, 2011; Madrid, 2020) and extends the findings examining the role of mood in voice behavior (Liu et al., 2015). Moreover, we extended the research about supervisors’ negative moods. It is known that supervisors’ negative mood influences employees’ assessment of their effectiveness (Lewis, 2000) and employees’ work performance and team atmosphere (Sy et al., 2005). We extend the work by demonstrating that in healthcare work settings, supervisors’ negative mood also influences employees’ extra-role behavior, and it even has different impacts on constructive and defensive voices.

Also, we contributed to the literature by explaining the underlying mechanism and the boundary conditions between supervisors’ negative moods and healthcare workers’ voice behavior. With the mediating role of healthcare workers’ negative mood, this study confirms the mood contagion between supervisors and employees in hospital units and supports the existing finding that negative mood inhibits voice as it conveys a tendency of risk avoidance (Fu et al., 2012). Moreover, this study extends the varieties of negative moods in impacting voice behavior: in previous studies, the negative moods being examined were mainly fear (e.g., Kish-Gephart et al., 2009) and guilt (e.g., Li et al., 2010). With dimensions of emotional intelligence as moderating variables, this study indicated that from the perspective of mood contagion, healthcare workers’ ability to perceive and control emotions also contributes to their voice behavior. It is worth noting that in our results, SEA, but not OEA, moderated the effects of supervisors’ negative mood on their negative mood and the indirect effect on their voice behavior. The reason OEA does not show the hypothesized moderation effect might be that for healthcare workers have stronger OEA: on the one hand, they can correctly perceive more emotion and mood from their supervisors, which will lead to more mood contagion; on the other hand, they are also able to understand why their supervisors have this kind of emotion or mood, which might decrease the degree of contagion. We believe these two factors might simultaneously influence the impact on the mood contagion process between supervisors and healthcare workers, thereby resulting in a non-significant moderation effect.

Methodologically, we investigate our proposed model within a high-risk and dynamic organizational context (hospital units). Thus, we connect with research on supervisors’ characteristics and subordinates’ voice within extreme environments (Weiss et al., 2018; Kee et al., 2021). Moreover, this study provides a more in-depth understanding of how employees’ proactive behavior (voice) is influenced in the pandemic background, which response to the amplifying importance of examining frontline healthcare workers’ behavior in this global pandemic (Hogan, 2020; Restubog et al., 2020).

For the practical implications, the association between supervisors’ negative mood and healthcare workers’ constructive voice and defensive voice suggests that healthcare workers’ supervisors should pay attention to their negative mood expression and regulation. This result would shed light on the monitoring systems that were applied in hospital units during the pandemic (Carter et al., 2021; Mitchell, 2021; Morris, 2021; Nemţeanu and Dabija, 2021). Moreover, with the significant moderating role of healthcare workers’ self-emotion appraisal ability influencing their voice behavior, it is suggested that in the hope of reducing being influenced by supervisors’ negative mood, training about noticing and recognizing their own emotions is also needed for healthcare workers.

This research also has some limitations. First, the cross-sectional nature of the research and the self-report measures prevent us from building causality inferences for the variables. It is recommended that future studies replicate the results by collecting healthcare workers’ voice behavior from other sources, such as supervisors’ or peers’ evaluations, and applying longitudinal designs to properly determine the magnitude and direction of the effect of supervisors’ mood on healthcare workers’ voice behavior. Additionally, collecting data via online questionnaires prevented us from reaching a broader range of participants, as those who did not have access to the link were excluded from this study. Future studies should employ additional methods to collect data.

Second, our studies were conducted only in Chinese hospital units, which requires careful interpretation when generalizing our results to culturally distinct contexts. Chinese people are in a more collectivistic culture and characterized by stronger interpersonal relationships (Hofstede, 2011). Consequently, supervisors’ negative moods might be of more concern for Chinese employees. In cultures that are more individualistic, the association of supervisors’ negative mood, healthcare workers’ negative mood, and voice behavior might be weaker than in the Chinese sample. We leave this proposition for future research.

## Conclusion

The present findings suggest that supervisors’ negative mood was positively associated with healthcare workers’ negative mood, which led to less constructive voice and more defensive voice. Moreover, this relationship depends on employees’ emotional intelligence: employees with a stronger ability to understand and express their own emotions may be less influenced by supervisors’ negative moods. Our results indicate that in hospital settings, supervisors should pay more attention to their negative mood expression and regulation; trainings about emotional intelligence, especially recognizing one’s own emotions are also needed.

## Data Availability Statement

The raw data supporting the conclusions of this article will be made available by the authors, without undue reservation.

## Author Contributions

PY: conceptualization. YC: writing—original draft preparation. YL: writing—review, editing, and data collection. SL: data analysis. All authors have read and agreed to the published version of the manuscript.

## Conflict of Interest

The authors declare that the research was conducted in the absence of any commercial or financial relationships that could be construed as a potential conflict of interest.

## Publisher’s Note

All claims expressed in this article are solely those of the authors and do not necessarily represent those of their affiliated organizations, or those of the publisher, the editors and the reviewers. Any product that may be evaluated in this article, or claim that may be made by its manufacturer, is not guaranteed or endorsed by the publisher.

## Footnotes

1 We did not find any significant differences in the results with and without the control variables. For simplicity, we report only the results without the control variables.

## References

[B1] Brislin R. W. (1980). “Cross-cultural research methods,” in *Environment and Culture*, eds Altman I. Rapoport A. Wohlwill J. F. (Boston, MA: Springer), 47–82.

[B2] Carter D. Kolencik J. Cug J. (2021). Smart internet of things-enabled mobile-based health monitoring systems and medical big data in COVID-19 telemedicine. *Am. J. Med. Res.* 8 20–29. 10.22381/ajmr8120212

[B3] Chen N. Y.-F. Crant J. M. Wang N. Kou Y. Qin Y. Yu J. (2021). When there is a will there is a way: the role of proactive personality in combating COVID-19. *J. Appl. Psychol.* 106, 199–213. 10.1037/apl0000865 33600195

[B4] Ciarrochi J. V. Chan A. Y. Caputi P. (2000). A critical evaluation of the emotional intelligence construct. *Pers. Individ. Diff.* 28 539–561. 10.1016/S0191-8869(99)00119-1

[B5] Clark A. (2021). Elevated anxiety symptoms, post-traumatic stress disorder, and moral trauma in COVID-19 frontline healthcare professionals. *Psychosociol. Issues Hum. Resour. Manag.* 9 89–98. 10.22381/pihrm9120219

[B6] Detert J. R. Burris E. R. (2007). Leadership behavior and employee voice: is the door really open? *Acad. Manag. J.* 50 869–884. 10.5465/amj.2007.26279183

[B7] Detert J. R. Treviño L. K. (2010). Speaking up to higher-ups: how supervisors and skip-level leaders influence employee voice. *Organ. Sci.* 21 249–270. 10.1287/orsc.1080.0405 19642375

[B8] Edmondson A. C. (2003). Speaking up in the operating room: how team leaders promote learning in interdisciplinary action teams. *J. Manag. Stud.* 40 1419–1452.

[B9] Frijda N. H. (1986). *The Emotions.* Cambridge, MA: Cambridge University Press.

[B10] Fu Q. Duan J. Tian X. (2012). The emotion mechanism of employee voice behavior: a new exploratory perspective. *Adv. Psychol. Sci.* 20 274–282. 10.3724/SP.J.1042.2012.00274

[B11] Glomb T. M. Hulin C. L. (1997). Anger and gender effects in observed supervisor-subordinate dyadic interactions. *Organ. Behav. Hum. Decis. Process.* 72 281–307. 10.1006/obhd.1997.2741 9606168

[B12] Hatfield E. Cacioppo J. T. Rapson R. L. (1993). *Emotional Contagion.* Cambridge, MA: Cambridge university press.

[B13] Hofstede G. (2011). Dimensionalizing cultures: the Hofstede model in context. *Online Read. Psychol. Cult.* 2 2307–2419.

[B14] Hogan M. J. (2020). Collaborative positive psychology: solidarity, mean- ing, resilience, well-being, and virtue in a time of crisis. *Int. Rev. Psychiatry* [Epub ahead of print]. 10.1080/09540261.2020.1778647 33427525

[B15] Hsiung H. H. Tsai W. C. (2017). The joint moderating effects of activated negative moods and group voice climate on the relationship between power distance orientation and employee voice behavior. *Appl. Psychol.* 66 487–514. 10.1111/apps.12096

[B16] Ilies R. Nahrgang J. D. Morgeson F. P. (2007). Leader-member exchange and citizenship behaviors: a meta-analysis. *J. Appl. Psychol.* 92 269–277. 10.1037/0021-9010.92.1.269 17227168

[B17] Kee K. van Wieringen M. Beersma B. (2021). The relational road to voice: how members of a low-status occupational group can develop voice behavior that transcends hierarchical levels. *J. Prof. Organ.* 8 253–272. 10.1093/jpo/joab011

[B18] Kish-Gephart J. J. Detert J. R. Treviño L. K. Edmondson A. C. (2009). Silenced by fear:: the nature, sources, and consequences of fear at work. *Res. Organ. Behav.* 29, 163–193. 10.1016/j.riob.2009.07.002

[B19] Lam C. F. Mayer D. M. (2014). When do employees speak up for their customers? A model of voice in a customer service context. *Pers. Psychol.* 67 637–666. 10.1111/peps.12050

[B20] Law K. S. Wong C. S. Song L. J. (2004). The construct and criterion validity of emotional intelligence and its potential utility for management studies. *J. Appl. Psychol.* 89 483–496. 10.1037/0021-9010.89.3.483 15161407

[B21] LePine J. A. Van Dyne L. (1998). Predicting voice behavior in work groups. *J. Appl. Psychol.* 83 853–868. 10.1037/0021-9010.83.6.853

[B22] Lewis K. M. (2000). When leaders display emotion: how followers respond to negative emotional expression of male and female leaders. *J. Organ. Behav.* 21 221–234. 10.1002/(sici)1099-1379(200003)21:2<221::aid-job36>3.0.co;2-0

[B23] Li A. N. Liao H. Tangirala S. Firth B. M. (2017). The content of the message matters: the differential effects of promotive and prohibitive team voice on team productivity and safety performance gains. *J. Appl. Psychol.* 102:1259. 10.1037/apl0000215 28358532

[B24] Li Y. Ahlstrom D. Ashkanasy N. M. (2010). A multilevel model of affect and organizational commitment. *Asia Pac. J. Manag.* 27 193–213. 10.1007/s10490-010-9193-9

[B25] Liu W. Tangirala S. Lam W. Chen Z. Jia R. T. Huang X. (2015). How and when peers’ positive mood influences employees’ voice. *J. Appl. Psychol.* 100:976. 10.1037/a0038066 25365730

[B26] Liu Y. Wei X. Chen X. (2011). The relationship among emotional intelligence, conflict management, and perceived cohension. *Sci. Res. Manag.* 32 88–96.

[B27] Lyons N. Birtus M. Cug J. (2021). Sustained psychological distress, acute depression, and emotional exhaustion in frontline medical staff and nurses working with COVID-19 patients. *Psychosociol. Issues Hum. Resour. Manag.* 9 99–108. 10.22381/pihrm91202110

[B28] Madrid H. P. (2020). Emotion regulation, positive affect, and promotive voice behavior at work. *Front. Psychol.* 11:1739. 10.3389/fpsyg.2020.01739 32765383 PMC7379878

[B29] Maynes T. D. Podsakoff P. M. (2014). Speaking more broadly: an examination of the nature, antecedents, and consequences of an expanded set of employee voice behaviors. *J. Appl. Psychol.* 99 87–112. 10.1037/a0034284 24041119

[B30] Mitchell K. (2021). Internet of things-enabled smart devices, healthcare body sensor networks, and online patient engagement in COVID-19 prevention, screening, and treatment. *Am. J. Med. Res.* 8 30–39. 10.22381/ajmr8120213

[B31] Mittal V. Ross W. T. Jr. (1998). The impact of positive and negative affect and issue framing on issue interpretation and risk taking. *Organ. Behav. Hum. Decis. Process.* 76 298–324. 10.1006/obhd.1998.2808 9878512

[B32] Morris K. (2021). Smart biomedical sensors, big healthcare data analytics, and virtual care technologies in monitoring, detection, and prevention of COVID-19. *Am. J. Med. Res.* 8 60–70. 10.22381/ajmr8120216

[B33] Morrison E. W. (2011). Employee voice behavior: integration and directions for future research. *Acad. Manag. Ann.* 5 373–412. 10.5465/19416520.2011.574506

[B34] Morrison E. W. (2014). Employee voice and silence. *Annu. Rev. Organ. Psychol. Organ. Behav.* 1 173–197. 10.1146/annurev-orgpsych-031413-091328

[B35] Muthén L. K. Muthén B. O. (2002). How to use a Monte Carlo study to decide on sample size and determine power. *Struct. Equ. Model.* 9 599–620. 10.1207/S15328007SEM0904_8 33486653

[B36] Nemeth C. J. (1997). Managing innovation: when less is more. *Calif. Manage. Rev.* 40, 59–74. 10.2307/41165922

[B37] Nembhard I. M. Edmondson A. C. (2006). Making it safe: the effects of leader inclusiveness and professional status on psychological safety and improvement efforts in health care teams. *J. Organ. Behav.* 27 941–966. 10.1002/job.413

[B38] Nemţeanu M. S. Dabija D. C. (2021). The influence of internal marketing and job satisfaction on task performance and counterproductive work behaviour in an Emergent Market during the COVID-19 pandemic. *Int. J. Environ. Res. Public Health* 18:3670. 10.3390/ijerph18073670 33915926 PMC8037227

[B39] Nemţeanu M. S. Dinu V. Dabija D. C. (2021). Job Insecurity, job instability and job satisfaction in the context of COVID 19 pandemic. *J. Competitiveness* 13 65–82. 10.7441/joc.2021.02.04

[B40] Neumann R. Strack F. (2000). “Mood contagion”: the automatic transfer of mood between persons. *J. Pers. Soc. Psychol.* 79 211–223. 10.1037//0022-3514.79.2.211 10948975

[B41] Platt C. (2021). Cognitive, emotional, and behavioral disorders in frontline medical staff and nurses working with COVID-19 patients. *Psychosociol. Issues Hum. Resour. Manag.* 9 7–16. 10.22381/pihrm9120211

[B42] Preacher K. J. Selig J. P. (2012). Advantages of Monte Carlo confidence intervals for indirect effects. *Commun. Methods Measures* 6 77–98. 10.1080/19312458.2012.679848

[B43] Restubog S. L. D. Ocampo A. C. G. Wang L. (2020). Taking control amidst the chaos: emotion regulation during the COVID-19 pandemic. *J. Vocation. Behav.* 119:103440. 10.1016/j.jvb.2020.103440 32390659 PMC7206430

[B44] Salovey P. Mayer J. D. (1990). Emotional intelligence. *Imagin. Cogn. Person.* 9 185–211. 10.2190/DUGG-P24E-52WK-6CDG 22612255

[B45] Stamu-O’Brien C. Carniciu S. Halvorsen E. Jafferany M. (2020). Psychological aspects of COVID-19. *J. Cosmetic Dermatol.* 19 2169–2173. 10.1111/jocd.13601 33439544

[B46] Sy T. Côté S. Saavedra R. (2005). The contagious leader: impact of the leader’s mood on the mood of group members, group affective tone, and group processes. *J. Appl. Psychol.* 90 295–305. 10.1037/0021-9010.90.2.295 15769239

[B47] Szczygiel D. D. Mikolajczak M. (2018). Emotional intelligence buffers the effects of negative emotions on job burnout in nursing. *Front. Psychol.* 9:2649. 10.3389/fpsyg.2018.02649 30627113 PMC6309155

[B48] Tangirala S. Ramanujam R. (2012). Ask and you shall hear (but not always): examining the relationship between manager consultation and employee voice. *Pers. Psychol.* 65 251–282. 10.1111/j.1744-6570.2012.01248.x

[B49] Van Kleef G. A. De Dreu C. K. Manstead A. S. (2010). “An interpersonal approach to emotion in social decision making: The emotions as social information model,” in *Advances in Experimental Social Psychology*, Vol. 42 ed. Zanna M. K. (Cambridge, MA: Academic Press), 45–96. 10.1016/s0065-2601(10)42002-x

[B50] Van Kleef G. A. Homan A. C. Beersma B. Van Knippenberg D. Van Knippenberg B. Damen F. (2009). Searing sentiment or cold calculation? The effects of leader emotional displays on team performance depend on follower epistemic motivation. *Acad. Manag. J.* 52 562–580. 10.5465/amj.2009.41331253

[B51] Weick K. E. Sutcliffe K. M. (2001). *Managing the Unexpected*, Vol. 9. San Francisco, CA: Jossey-Bass.

[B52] Weiss M. Kolbe M. Grote G. Spahn D. R. Grande B. (2018). We can do it! Inclusive leader language promotes voice behavior in multi-professional teams. *Leadership Q.* 29 389–402. 10.1016/j.leaqua.2017.09.002

